# Photoperiod Following Inoculation of Arabidopsis with *Pyricularia oryzae* (syn. *Magnaporthe oryzae*) Influences on the Plant–Pathogen Interaction

**DOI:** 10.3390/ijms22095004

**Published:** 2021-05-08

**Authors:** Sayaka Shimizu, Yuri Yamauchi, Atsushi Ishikawa

**Affiliations:** Department of Bioscience and Biotechnology, Fukui Prefectural University, Fukui 910-1195, Japan; s1521024@g.fpu.ac.jp (S.S.); s1421046@g.fpu.ac.jp (Y.Y.)

**Keywords:** nonhost resistance, photoperiod, Arabidopsis, *Pyricularia oryzae*

## Abstract

In plant–pathogen interactions, a proper light environment affects the establishment of defense responses in plants. In our previous experiments, we found that nonhost resistance (NHR) to *Pyricularia oryzae* Cav. in *Arabidopsis thaliana* (L.) Heynh. (Arabidopsis), in diurnal conditions, varies with the inoculation time. Moreover, we indicated that the circadian clock plays an important role in regulating time-of-day differences in NHR to *P. oryzae* in Arabidopsis. However, the involvement of photoperiod in regulating NHR was still not understood. To determine the photoperiod role, we performed the experiments in continuous light and darkness during the early Arabidopsis–*P. oryzae* interaction. We found that the light period after the inoculation in the evening enhanced the resistance to penetration. However, the dark period after the inoculation in the morning suppressed the penetration resistance. Furthermore, the genetic analysis indicated that jasmonic acid, reactive oxygen species, and tryptophan-derived metabolite(s) contribute to the photoperiod regulation of NHR in Arabidopsis. The present results denote that photoperiod plays an important role in regulating time-of-day differences in NHR to *P. oryzae* in Arabidopsis.

## 1. Introduction

Light directly regulates plant physiology and development as well as affects many plant responses to the environment, including defense responses in plant–pathogen interactions [1,2,3]. Light can regulate plant responses by two mechanisms: photosynthesis and photoreceptor signaling in plants [1,2,3]. Photosynthesis contributes to the supply of reducing equivalents and energy for metabolite production. Plant photoreceptors perceive surrounding red/far-red (phytochromes), blue/UV-A (cryptochromes, phototropins), and UV-B light (UVR8) [3,4]. Previous studies in plants showed that resistance responses require light [1,2,3], and in many plant–pathogen interactions, resistance responses are weakened in the dark [5,6,7,8]. It has recently been shown that chloroplast is involved in plant immunity [9]. Therefore, light has effects on defense responses through the regulation of chloroplast function. Furthermore, light affects pathogenesis through the regulation of pathogen growth and development [1]; as an example, light inhibits spore germination and germ tubes’ growth in many plant fungi [10,11,12,13,14].

Disease resistance shown by a plant species to all genetic variants of a pathogen species that have not adapted to it is called nonhost resistance (NHR) [15,16]. Several penetration-resistance genes of *Arabidopsis thaliana* (L.) Heynh. (Arabidopsis) mutants with compromised NHR to non-adapted powdery mildew *Blumeria graminis* f. sp. *hordei* (*Bgh*) have been identified: *penetration 1* (*pen1*), *pen2*, and *pen3* [17,18,19,20,21,22,23]. *PEN1* encodes a plasma membrane-anchored syntaxin [17], *PEN2* encodes a myrosinase involved in indole glucosinolate (IG) metabolism used in defense responses [18], and *PEN3* encodes an ATP-binding cassette transporter [19,20]. The PEN2 hydrolyzes IGs and produces broad-spectrum toxic compounds with antifungal activity in Arabidopsis [21], and PEN3 transports the bioactive metabolite(s) of the PEN2 pathway and camalexin, which is synthesized through CYP71B15 (PAD3) [22] into the apoplast at pathogen contact sites [20,23].

Glucosinolates (GSs) are secondary metabolites with defensive function in members of the order Brassicales [24]. IGs are derived from tryptophan (Trp), and the first step in IG biosynthesis is catalyzed by CYP79B2 and CYP79B3 [24]. These two P450 monooxygenases convert Trp into indole-3-acetaldoxime (IAOx) [25]. IAOx is a precursor of the IG, camalexin, and indole-3-carboxylic acid derivatives. These indole-type metabolites act in defense responses in Arabidopsis. In fact, *cyp79B2 cyp79B3* mutant plants are highly susceptible to many plant fungal pathogens [26,27,28].

Rice blast caused by *Pyricularia oryzae* Cav. (syn. *Magnaporthe oryzae* Couch.) is a devastating disease of rice. The mechanisms of resistance to *P. oryzae* have been extensively studied, and the rice–*P. oryzae* pathosystem has become a model for plant–microbe interaction studies [29,30]. We have previously investigated the mechanisms of NHR to *P. oryzae* in Arabidopsis and found a suite of genes involved in NHR to *P. oryzae in* Arabidopsis [31,32]. For example, we have shown that *pen2-1 mol2 pmr5 gl1 NahG* (*pm5gN*) plants were severely compromised for NHR to *P. oryzae*. Those findings indicate that *PEN2, MILDEW RESISTANCE LOCUS O 2* (*MLO2*)*,* and *POWDERY MILDEW RESISTANCE 5* (*PMR5*) are involved in the establishment of NHR to *P. oryzae* in Arabidopsis [32]. In addition, we have examined the involvement of other defense-related genes in NHR to *P. oryzae* in Arabidopsis, including *PHYTOALEXIN DEFICIENT 1* (*PAD1*), *PHYTOALEXIN DEFICIENT 3* (*PAD3*), *JASMONATE RESISTANT 1* (*JAR1*), *SUPPRESSOR OF BIR1* (*SOBIR1*), and *ARABIDOPSIS RESPIRATORY BURST OXIDASE HOMOLOGUES* (*AtRbohD* and *AtRbohF*) and found that some of them contribute to NHR to *P. oryzae* in Arabidopsis [31,33,34].

In a previous report, we found that NHR to *P. oryzae* in Arabidopsis varies with inoculation time in diurnal conditions [35]. These prior results showed a lower resistance after inoculation in the evening rather than in the morning. In conclusion, the plant’s internal clock, its photoperiod, or both play an important role in regulating time-of-day differences in NHR to *P. oryzae* in Arabidopsis. Indeed, we have shown that the circadian clock component CCA1 contributes to time-of-day-dependent penetration resistance and that another component LHY regulates post-penetration resistance [36]. However, the involvement of photoperiod in regulating NHR is still not understood. In rice–*P. oryzae* interaction, the effect of light and darkness on the infection of rice blast fungus was investigated as early as the 1930s [37] and references cited therein. Their results suggested that light tends to inhibit *P. oryzae* infection of rice plants [37]. In the present research, we performed the daytime experiment both in continuous light and in continuous darkness during the early Arabidopsis—*P. oryzae* interaction to discriminate between circadian regulation and photoperiod regulation.

## 2. Results

**Figure 1:**
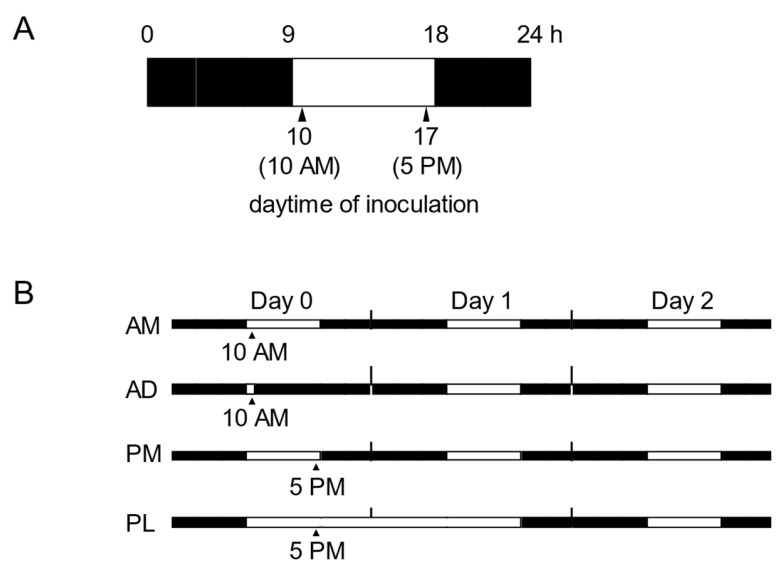
Time scheme used in this study. (**A**) Illustration of daytimes of *P. oryzae* inoculation. Arabidopsis plants were inoculated at 10:00 a.m. and 5:00 p.m. (**B**) Illustration of light–dark conditions and inoculation times during the experiments. The illustration depicts light regime and inoculation times until day 2. Inoculation was done on day 0, and leaves were harvested on day 3. AM: Arabidopsis plants inoculated at 10:00 a.m.; AD: Arabidopsis plants inoculated at 10:00 a.m. and transferred to continuous darkness until entering the light period in the growth chamber; PM: Arabidopsis plants inoculated at 5:00 p.m.; PL: Arabidopsis plants inoculated at 5:00 p.m. and transferred to continuous lightness until entering the dark period in the growth chamber. White and black boxes correspond to light and dark periods, respectively. Arrowheads and bottom numbers indicate the inoculation times.

In our experiments, Arabidopsis plants were grown under short-day conditions (9:15 L:D) at 22 °C (100 umol m^−2^s^−1^ fluorescent illumination) in a growth chamber. To study NHR in Arabidopsis, we inoculated plants in the morning (10:00 a.m.) and in the evening (5:00 p.m.) (Figure 1A). In this study, to examine the effect of light/dark period following inoculation on NHR to *P. oryzae* in Arabidopsis, we changed the photoperiod after inoculation in the morning and in the evening, respectively (Figure 1B).

### 2.1. The Light Period Following Evening Inoculation Enhances NHR to P. oryzae in Arabidopsis

**Figure 2:**
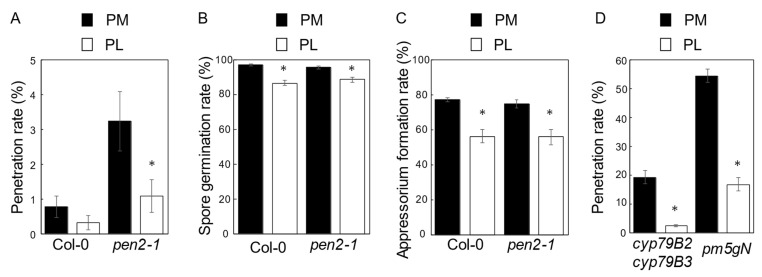
Quantitative analyses of penetration resistance to *P. oryzae* in Arabidopsis plants inoculated in the evening. (**A**) Mean frequency of *P. oryzae* penetration into Col-0 (wild-type) and *pen2-1* plants at 72 h post-inoculation (hpi) expressed as a percentage of the total number of infection sites. (**B**) Spore germination rates on Col-0 (wild-type) and *pen2-1* leaves at 72 hpi. Rates are expressed as a percentage of the total number of conidia. (**C**) Appressorium formation rates on Col-0 (wild-type) and *pen2-1* leaves at 72 hpi. Rates are expressed as a percentage of the total number of conidia. (**D**) Mean frequency of *P. oryzae* penetration into *cyp79B2 cyp79B3* and *pen2-1 mol2 pmr5 gl1 NahG* (*pm5gN*) (*pm5gN*) plants at 72 hpi expressed as a percentage of the total number of infection sites. Values are expressed as mean ± standard error from three independent experiments, each containing six biological replicates (*n* = 18). Black bar: PM inoculation; white bar: PL inoculation. Values marked with asterisks (PL inoculation) are significantly different from respective PM inoculation-controls (Student’s *t*-test; *p* < 0.05).

**Figure 3:**
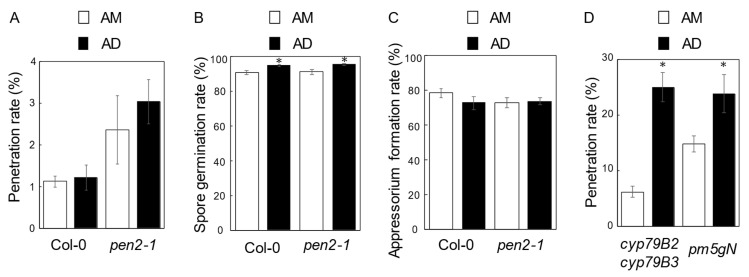
Quantitative analyses of penetration resistance to *P. oryzae* in Arabidopsis plants inoculated in the morning. (**A**) Mean frequency of *P. oryzae* penetration into Col-0 (wild-type) and *pen2-1* plants at 72 hpi expressed as a percentage of the total number of infection sites. (**B**) Spore germination rates on Col-0 (wild-type) and *pen2-1* leaves at 72 hpi. Rates are expressed as a percentage of the total number of conidia. (**C**) Appressorium formation rates on Col-0 (wild-type) and *pen2-1* leaves at 72 hpi. Rates are expressed as a percentage of the total number of conidia. (**D**) Mean frequency of *P. oryzae* penetration into *cyp79B2 cyp79B3* and *pm5gN* plants at 72 hpi expressed as a percentage of the total number of infection sites. Values are expressed as mean ± standard error from three independent experiments, each containing six biological replicates (*n* = 18). Black bar: AD inoculation; white bar: AM inoculation. Values marked with asterisks (AD inoculation) are significantly different from respective AM inoculation-controls (Student’s *t*-test; *p* < 0.05).

The *pen2-1* plants inoculated in the evening (5:00 p.m.) showed a decreased resistance compared with those inoculated in the morning (10:00 a.m.) (Figure 2A and Figure 3A) [35,36]. This result suggests that a dark period following the evening inoculation would have a suppressive impact on NHR. To investigate this hypothesis, plants were inoculated in the evening and kept under different conditions. We compared the penetration resistance in plants that were maintained under normal photoperiodic conditions (PM, Figure 1B) with those that were transferred to continuous light conditions until entering the dark period in the growth chamber (PL, Figure 1B). Col-0 and *pen2-1* plants were inoculated with *P. oryzae*. Then, the leaves of infected plants 72 h post-inoculation (hpi) were harvested and examined microscopically. The accumulation of autofluorescent compounds in the challenged epidermal cell was used as a marker for penetration of *P. oryzae*. Cell entry was quantified in six leaves from six independent plants. A minimum of 100 infection sites were inspected per leaf. Upon inoculation onto the Col-0 plants in the evening, the penetration rate of *P. oryzae* into leaves of Col-0 plants in the PM condition was not different from that of Col-0 plants under the PL condition (Figure 2A). In contrast, the penetration rate of *P. oryzae* into leaves of *pen2-1* plants in the PM condition was significantly higher than that of *pen2-1* plants in the PL condition (Figure 2A).

To determine whether the light/dark period following inoculation in the evening affected spore germination and appressorium formation of *P. oryzae*, we examined the spore germination rate and the appressorium formation rate at 72 hpi on Col-0 and *pen2-1* plants’ leaves. To quantify the rates, we examined the fungal sporelings on six leaves from six independent plants per experiment and genotype (minimum of 100 conidia/leaf evaluated). The spore germination rate on Col-0 plants in the PL condition was significantly lower than the rate on Col-0 plants in the PM condition (Figure 2B). Moreover, the appressorium formation rate on Col-0 plants under the PL condition was significantly lower than the rate on Col-0 plants in the PM condition (Figure 2C). Furthermore, spore germination rate and appressorium formation rate on *pen2-1* plants under the PL condition were significantly lower than the PM condition (Figure 2B,C).

To more deeply examine the effect of the light condition on NHR, we investigated penetration resistance in Arabidopsis *cyp79B2 cyp79B3* and *pen2-1 mol2 pmr5 gl1 NahG* (*pm5gN*) plants. In these mutant plants, the penetration rates were significantly higher than those of *pen2-1* plants in the PM condition, suggesting the severely compromised NHR in these plants. Nonetheless, the penetration rates under the PL condition were significantly lower than those of the PM condition (Figure 2D). These results indicate that the light period after the evening inoculation had significant suppressive effects on spore germination and appressorium formation of *P. oryzae*, leading to enhanced penetration resistance in the PL condition in Arabidopsis. Thus, the enhanced NHR in the PL condition is primarily related to the effect of light on *P. oryzae*.

### 2.2. The Dark Period Following the Morning Inoculation Suppresses NHR to P. oryzae in Arabidopsis

The *pen2-1* plants inoculated in the morning showed enhanced resistance compared with the plants evening inoculated (Figure 2A and Figure 3A) [35,36]. This result suggests that the light period after morning inoculation would have a positive impact on NHR. To pursue this hypothesis, plants were inoculated in the morning, and penetration resistance in plants kept under different conditions was compared. Plants were maintained under normal photoperiodic conditions (AM, Figure 1B), and the others were transferred to continuous darkness until entering the light period in the growth chamber (AD, Figure 1B). Upon inoculation onto the Col-0 plants in the morning, the penetration rate of *P. oryzae* into leaves of Col-0 plants in the AD condition was not different from that of Col-0 plants in the AM condition (Figure 3A). The penetration rate of *P. oryzae* into leaves of *pen2-1* plants in the AD condition was slightly higher, not significantly, than that of *pen2-1* plants in the AM condition (Figure 3A).

Next, to determine whether the light/dark period following inoculation in the morning affected spore germination and appressorium formation of *P. oryzae*, we examined the spore germination rates and the appressorium formation rates at 72 hpi on leaves of Col-0 and *pen2-1* plants. The spore germination rates on Col-0 and *pen2-1* plants in the AD condition were significantly higher than those of plants in the AM condition (Figure 3B). However, the appressorium formation rates on Col-0 and *pen2-1* plants in the AD condition were not different from those under the AM condition (Figure 3C). These results indicate that the dark period following the inoculation in the morning had little effect on the appressorium formation rates of *P. oryzae*.

Next, we investigated penetration resistance in Arabidopsis *cyp79B2 cyp79B3* and *pm5gN* mutant plants (Figure 3D). In these mutants, the penetration rates were higher than that of *pen2-1* plants in the AM and AD conditions, suggesting the severely compromised NHR in these mutants in the morning inoculation. Moreover, the penetration rates in these mutants in the AD condition were significantly higher than those of the AM condition (Figure 3D and Figure S1). These results point out that the dark period after morning inoculation had significant suppressive effects on the penetration resistance in the AD condition. The suppression is primarily due to the dark condition’s effect on Arabidopsis but not on *P. oryzae*.

Our results indicate that the photoperiod after morning inoculation and evening inoculation would impact the Arabidopsis—*P. oryzae* interaction outcome in two ways. First, in the PL condition, the light period following the evening inoculation suppressed the proper development of *P. oryzae*, leading to an enhanced NHR in Arabidopsis (Figure 2). Second, in the AD condition, the dark period after morning inoculation had a suppression effect on penetration resistance in Arabidopsis (Figure 3D).

### 2.3. Genes Controlling Photoperiod-Dependent NHR to P. oryzae in Arabidopsis

To identify genes responsible for controlling photoperiod-dependent NHR to *P. oryzae* in Arabidopsis, we inoculated several *pen2-1* double mutants (Table S1), which we had studied so far, under AM and AD conditions and examined their penetration resistance. These *pen2-1* double mutants were grouped into the following three categories according to their functions: (1) Jasmonic acid (JA)-related and other defense-related genes (*PAD1*, *JAR1*, *PMR5*, and *SOBIR1*); (2) Reactive oxygen species (ROS)-related genes (*AtRbohD* and *AtRbohF*); and (3) Tryptophan (Trp)-derived metabolites-related genes (*PAD3*, *PEN3*, *CYP79B2*, and *CYP79B3*).

**Figure 4:**
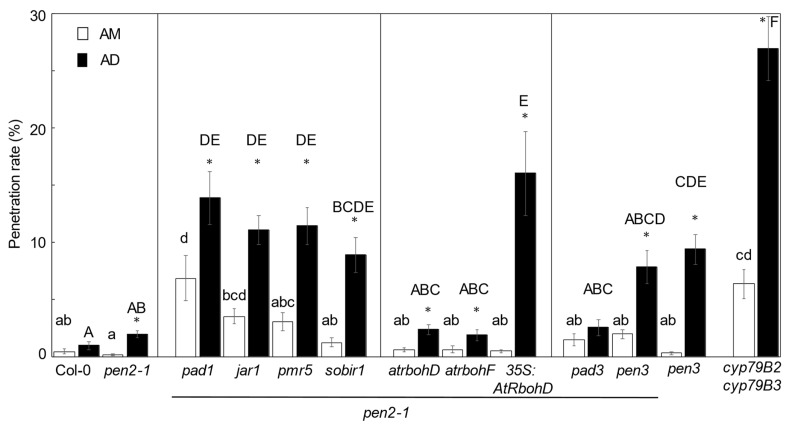
Penetration resistance to *P. oryzae* in *pen2-1* double mutants inoculated in the morning. Mean frequency of *P. oryzae* penetration into Col-0 (wild-type), *pen2-1, pen2-1* double mutants, *pen3*, and *cyp79B2 cyp79B3* plants at 72 hpi expressed as a percentage of the total number of infection sites. Values are expressed as mean ± standard error from three independent experiments, each containing six biological replicates (*n* = 18). Black bar: AD inoculation; white bar: AM inoculation. Significantly different statistical groups of genotypes indicated by the analyses of variance (Tukey’s test; *p* < 0.05) are shown with lowercase (AM inoculation) and uppercase (AD inoculation) letters. Values marked with asterisks (AD inoculation) are significantly different from respective AM inoculation-controls (Student’s *t*-test; *p* < 0.05).

First, we compared the penetration rates of *P. oryzae* in leaves of all mutants between AM and AD conditions. In these mutants, except for *pen2-1 pad3* mutant, the penetration rates of *P. oryzae* in leaves in the AD condition were significantly higher than those in the AM condition (Figure 4). This result suggests that NHR to *P. oryzae* in many *pen2-1* double mutants was significantly suppressed in the AD condition compared with that in the AM condition. Second, we compared the penetration rates of *P. oryzae* in leaves of all *pen2-1* double mutants with those in leaves of control Col-0 and *pen2-1* plants in the AM condition. We found that the penetration rates in *pen2-1 pad1* and *cyp79B2 cyp79B3* mutants were significantly higher than those in leaves of control plants (Figure 4). This result indicates that PAD1, CYP79B2, and CYP79B3 are involved in NHR establishment in the AM condition. Third, we examined the penetration rates of *P. oryzae* in leaves of group 1 plants in the AD condition. In these mutants, except for *pen2-1 sobir1*, the penetration rates of *P. oryzae* into leaves were significantly higher than those in leaves of *pen2-1* plants (Figure 4). This result indicates that PAD1, JAR1, and PMR5, but not SOBIR1, are involved in NHR establishment in the AD condition. Fourth, to study the ROS effect on NHR under the AD condition, we investigated group 2 plants. We found that only in *pen2-1 35S:AtRbohD* plants was the penetration rate of *P. oryzae* in leaves significantly higher than that in leaves of *pen2-1* plants in the AD condition (Figure 4). However, we did not detect any differences between *pen2-1 atrboh* mutants and control *pen2-1* plants (Figure 4). These results indicate that overexpressed *AtRbohD*-dependent ROS production influences NHR in the AD condition. Fifth, to study the effect of Trp-derived metabolites on NHR under AD condition, we examined group 3 plants. Among them, only in *pen3* plants was the penetration rate of *P. oryzae* in leaves significantly higher than that in the leaves of *pen2-1* plants under the AD condition (Figure 4). This result indicates that the PEN3-dependent pathway is required for NHR under the AD condition. Further, in *cyp79B2 cyp79B3* plants, the penetration rate of *P. oryzae* in the leaves was significantly higher than that in the leaves of *pen2-1* plants under the AD condition (Figure 4). The same result is observed in Figure 3D. These results imply that CYP79B2 and CYP79B3-dependent Trp-derived metabolite(s) is required for NHR establishment under the AD condition.

## 3. Discussion

We previously found that NHR to *P. oryzae* in Arabidopsis varies with the inoculation time in diurnal conditions and shows that the circadian clock regulates NHR to *P. oryzae* in Arabidopsis [35,36]. In this study, we investigated the photoperiod influence during early plant–pathogen interactions on NHR in Arabidopsis. We found that the duration of the light/dark period just after inoculation impacts the Arabidopsis—*P. oryzae* interaction. The presence of light after inoculation showed positive effects on NHR through repression of fungal development. The absence of light during the early interaction period entails suppressed resistance through plant resistance responses’ suppression. From the analysis of the results, we concluded that the light/dark period after inoculation, in addition to the circadian clock, affects Arabidopsis resistance. Indeed, a light period of a certain length following pathogen inoculation has been reported as a prerequisite for optimal defense in some plant–pathogen interactions [1,2]. As an example, in an incompatible interaction between rice and *Xanthomonsa oryzae*, a minimum of 8 h of light after pathogen inoculation was required for hypersensitive response (HR) and restriction of pathogen growth [5]. Furthermore, in Arabidopsis–Turnip crinkle virus interaction, >6 h of light after infection was required for HR and *PR-1* expression [8]. These data indicate that photoperiod during early plant–pathogen interactions plays an important role in the establishment of defense responses.

In our previous report, we compared the appressorium formation rates of *P. oryzae* after the morning and the evening inoculation and discovered that the inoculation time had little effect on the appressorium formation rates of *P. oryzae* [35] (see Figure 2 and Figure 3). Thus, the differences in outcomes of the interaction between AM and PM conditions are primarily due to the inoculation time effect on Arabidopsis but not on the pathogen. However, in this study, we found that the appressorium formation rate of *P. oryzae* in the PL condition was significantly reduced compared with the PM condition (Figure 2). The light period continued until entering the dark period in the growth chamber in the PL condition. This result suggests that a prolonged light period (25 h) following pathogen inoculation suppresses the appressorium formation of *P. oryzae*. Future studies are needed to define the effect of light on fungal development.

In plants, the dark period would suppress the defense responses through a reduction in photosynthesis and following carbon starvation or light signal suppression. Indeed, the *phyA phyB phyC* mutant rice plants are more susceptible to *P. oryzae* compared with wild-type plants, indicating that phytochromes-dependent light perception and associated signaling pathways are required for resistance to *P. oryzae* in rice [38]. Moreover, phytochrome signaling controls systemic acquired resistance in Arabidopsis [39]. Furthermore, light/dark conditions affect defense responses through the regulation of chloroplast function. One of the reasons is that the synthetic pathways for some plant hormones, such as JA, salicylic acid (SA), and abscisic acid, depend on plastids [9]. Another reason is that ROS are produced from chloroplasts during stress conditions [9]. Thus, plant hormones and ROS are candidate factors that control NHR in a light/dark-dependent fashion. We found that JAR1 and PAD1 are involved in the regulation of NHR in the AD condition (Figure 4). JAR1 encodes a jasmonate-amido synthetase, which is a member of the GH3 family of proteins and catalyzes the formation of a biologically active jasmonyl-isoleucine conjugate [40]. PAD1 is involved in the JA signaling pathway [41]. Thus, a JA-mediated pathway would be required for establishing NHR in the AD condition. Moreover, we found that the overexpression of *AtRbohD* in *pen2-1* plants led to the suppression of NHR in the AD condition (Figure 4). Arabidopsis AtRbohD and AtRbohF are NADPH oxidases, identified as key components of plant defenses [42,43]. Thus, ROS might act as a negative regulator of NHR in the AD condition. However, we did not detect differences in penetration rates between *pen2-1 atrboh* mutants and *pen2-1* plants in AM and AD conditions. Further study will be needed to reveal the function of ROS in the establishment of NHR.

SOBIR1 functions as a coreceptor to control receptor-like kinase or receptor-like protein signaling networks and regulates plant immunity [44]. We did not detect significant differences in penetration rates between *pen2-1 sobir1* mutant and *pen2-1* mutant plants under AM and AD conditions. This result suggests that SOBIR1-dependent signal transduction does not play a major role in NHR establishment under these conditions.

In *pen2-1 pen3* plants, the penetration rate of *P. oryzae* into leaves was slightly higher, not significantly, than in *pen2-1* plants under AD condition; however, the penetration rate of *P. oryzae* into *pen3* leaves was significantly higher than into leaves of Col-0 plants in the AD condition. If PEN2 and PEN3 work in concert in Arabidopsis–*P. oryzae* interaction, then it is expected that *pen2-1*, *pen3*, and *pen2-1 pen3* have the same phenotype. However, only *pen3* plants showed significantly compromised penetration resistance compared with control plants in the AD condition. This result suggests that the PEN3-dependent pathway, but not the PEN2-dependent pathway, is required for establishing proper NHR in the AD condition. It is assumed that the substrate(s) of PEN3 should not be exported in *pen3* plants and that the failure would have a suppressive effect on NHR in the AD condition. As PEN3 functions redundantly with PLEIOTROPIC DRUG RESISTANCE (PDR) 12 in the export of camalexin in Arabidopsis [23], it is possible that camalexin is involved in NHR in the AD condition. However, although CYP71B15 (PAD3) catalyzes the rate-limiting steps of camalexin synthesis from IAOx [22], the penetration rate of *P. oryzae* into leaves of *pen2-1 pad3* plants was not different from that of *pen2-1* plants under AM and AD conditions (Figure 4). This result suggests that camalexin is not involved in the establishment of NHR in these conditions. Moreover, although PEN3 is assumed to export CYP79B2 CYP79B3-dependent Trp-derived metabolite(s), the penetration rate of *P. oryzae* into leaves of *cyp79B2 cyp79B3* plants was significantly higher than that of *pen3* plants in the AD condition (Figure 4). This result suggests that the CYP79B2 CYP79B3-dependent but PEN3-independent pathway would also contribute to the establishment of NHR under AD condition.

We demonstrated that NHR to *P. oryzae* in Arabidopsis varies with the inoculation time in diurnal conditions and that the circadian clock plays an important role in regulating time-of-day differences in NHR. In this study, we show that NHR to *P. oryzae* in Arabidopsis is correlated positively with the light period during the early Arabidopsis—*P. oryzae* interaction and that the photoperiod plays a significant role in regulating time-of-day differences in NHR to *P. oryzae* in Arabidopsis. The photoperiod might affect NHR through the regulation of JA, ROS, and Trp-derived metabolite(s) in Arabidopsis.

NHR is the most robust and durable resistance in plants. Future studies are needed to understand the genetic and mechanistic mechanisms, including the interaction between the circadian clock and photoperiod of NHR to *P. oryzae* in Arabidopsis. Thus, the mechanisms findings can be used to improve plants’ defense responses and contribute to the increased productivity of crop plants in the future.

## 4. Materials and Methods

### 4.1. Plant Materials and Growth Conditions

The plants used in this study were Col-0 (wild-type), pen2-1 [18], pen2-1 pad1 [31], pen2-1 jar1 [31], pen2-1 pmr5 [31], pen2-1 sobir1 [45], pen2-1 atrbohd [33], pen2-1 atrbohf [33], pen2-1 35S:AtRbohD [43], pen3 (pdr8-1) [31], pen2-1 pen3, cyp79B2 cyp79B3 [25], and pen2-1 mol2 pmr5 gl1 NahG (pm5gN) [32], which are all on the Col-0 background. Arabidopsis mutants used to construct pen2-1 double mutants are listed in Table S1. We used pen2-1, pen3, and 35S:AtRbohD plants in the cross to generate pen2-1 double mutant plants.

Arabidopsis plants were grown on Murashige and Skoog plates in a growth room for three weeks in short-day length conditions (9:15 L:D) at 22 °C (in 100 umol m^−2^sec^−1^ fluorescent illumination). Then, the plants were transferred to soil and grown in a growth chamber for 4 weeks, where they continued to grow in short-day length conditions (9:15 L:D) at 22 °C (in 100 umol m^−2^s^−1^ fluorescent illumination).

### 4.2. Pathogen Inoculation and Analysis

We incubated *P. oryzae* isolate Hoku 1 (race 007) on an oatmeal agar media at 25 °C. Then, we inoculated Arabidopsis with *P. oryzae* by applying 5 uL droplets (5 × 10^4^ spores/mL) of *P. oryzae* onto leaves (leaf number 8) of rosettes on Arabidopsis (i.e., leaves numbered from oldest to youngest) in the morning and the evening. Then, inoculated plants were maintained in a growth chamber with saturating humidity in short-day conditions (9 h:15 h light:dark) at 22 °C (in 100 umol m^−^^2^s^−1^ fluorescent illumination). Inoculated leaves were harvested at 72 h post-inoculation (hpi).

To quantify the spore germination rates and the appressorium formation rates of *P. oryzae*, we examined the fungal sporelings. This examination was performed on six leaves from six independent plants per experiment and genotype (minimum of 100 conidia/leaf evaluated). Experiments were repeated thrice.

To quantify cell penetration, we examined germinated fungal sporelings that had developed appressoria (six leaves from six independent plants per experiment and genotype). We evaluated a minimum of 100 appressoria/leaves. We detected successful penetration of *P. oryzae* by observing autofluorescence or hyphal elongation at infection sites with fluorescence and bright-field microscopy. Each plant genotype was quantified in three independent experiments.

## Data Availability

All data supporting this study are available in this paper and in its supplementary data published online.

## Supplementary Materials

The following are available online at https://www.mdpi.com/, Table S1: List of Arabidopsis mutants used to construct *pen2-1* double mutants, Figure S1: Plant response to *P. oryzae* infection.
