# Supplementary material for: Photoperiod Following Inoculation of Arabidopsis with Pyricularia oryzae (syn. Magnaporthe oryzae) Influences on the Plant–Pathogen Interaction
Source: Int J Mol Sci. 2021 May 8;22(9):5004. doi: 10.3390/ijms22095004 (PMC8125946; doi:10.3390/ijms22095004)
Supplement: Supplementary file 1 [file ijms-22-05004-s001.zip › ijms-1191829-supplementary.pdf]

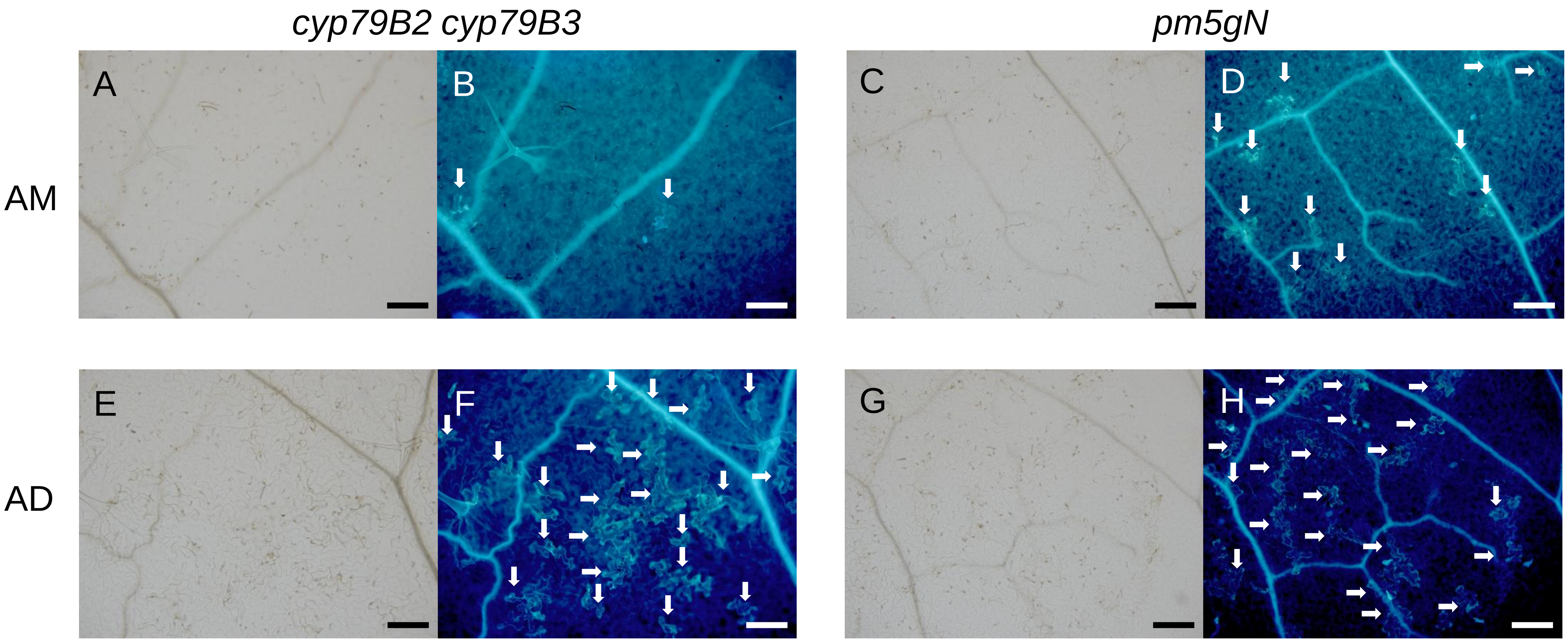

Figure S1. Plant response to *P. oryzae* infection.
Light microscopic view of infection sites of *cyp79B2 cyp79B3* (A, E) and *pm5gN* (C, G) plants at 72 hpi. Cell death associated autofluorescence at infection sites of *cyp79B2 cyp79B3* (B, F) and *pm5gN* (D, H) plants as visualized by fluorescence microscopy. AM inoculation (A-D). AD inoculation (E-H). White arrow indicates a penetrated epidermal cell. Bar, 0.2 mm.

Figure S1

Table S1

List of Arabidopsis mutants used to construct *pen2-1* double mutants

Mutant	Function	Reference
pen2-1	myrosinase	1
pad1	JA signaling	2
jar1	JA signaling	3
pmr5	putative pectin acetyltransferase	4
sobir1	pattern recognition receptor	5
atrbohd	NADPH oxidase	6
atrbohf	NADPH oxidase	6
35S:AtRbohD	NADPH oxidase	7
pad3	camalexin synthesis	8
pen3	ABC transporter	9,10

1. Lipka, V., J. Dittgen, P. Bednarek, R. Bhat, M. Wiermer, M. Stein, J. Landtag, W. Brandt, S. Rosahl, D. Scheel, et al., *Pre- and postinvasion defenses both contribute to nonhost resistance in Arabidopsis.* Science, 2005. **310**(5751): p. 1180-3.

2. Glazebrook, J. and F.M. Ausubel, *Isolation of phytoalexin-deficient mutants of Arabidopsis thaliana and characterization of their interactions with bacterial pathogens.* Proc Natl Acad Sci U S A, 1994. **91**(19): p. 8955-9.

3. Staswick, P.E., W. Su, and S.H. Howell, *Methyl jasmonate inhibition of root growth and induction of a leaf protein are decreased in an Arabidopsis thaliana mutant.* Proc Natl Acad Sci U S A, 1992. **89**(15): p. 6837-40.

4. Vogel, J.P., T.K. Raab, C.R. Somerville, and S.C. Somerville, *Mutations in PMR5 result in powdery mildew resistance and altered cell wall composition.* Plant J, 2004. **40**(6): p. 968-78.

5. Gao, M., X. Wang, D. Wang, F. Xu, X. Ding, Z. Zhang, D. Bi, Y.T. Cheng, S. Chen, X. Li, et al., *Regulation of cell death and innate immunity by two receptor-like kinases in Arabidopsis.* Cell Host Microbe, 2009. **6**(1): p. 34-44.

6. Torres, M.A., J.L. Dangl, and J.D.G. Jones, *Arabidopsis gp91(phox) homologues AtrbohD and AtrbohF are required for accumulation of reactive oxygen intermediates in the plant defense response.* Proceedings of the National Academy of Sciences of the United States of America, 2002. **99**(1): p. 517-522.

7. Torres, M.A., J.D.G. Jones, and J.L. Dangl, *Pathogen-induced, NADPH oxidase-derived reactive oxygen intermediates suppress spread of cell death in Arabidopsis thaliana.* Nature Genetics, 2005. **37**(10): p. 1130-1134.

8. Zhou, N., T.L. Tootle, and J. Glazebrook, *Arabidopsis PAD3, a gene required for camalexin biosynthesis, encodes a putative cytochrome P450 monooxygenase.* Plant Cell, 1999. **11**(12): p. 2419-2428.

9. Kobae, Y., T. Sekino, H. Yoshioka, T. Nakagawa, E. Martinoia, and M. Maeshima, *Loss of AtPDR8, a plasma membrane ABC transporter of Arabidopsis thaliana, causes hypersensitive cell death upon pathogen infection.* Plant Cell Physiol, 2006. **47**(3): p. 309-18.

10. Stein, M., J. Dittgen, C. Sanchez-Rodriguez, B.H. Hou, A. Molina, P. Schulze-Lefert, V. Lipka, and S. Somerville, *Arabidopsis PEN3/PDR8, an ATP binding cassette transporter, contributes to nonhost resistance to inappropriate pathogens that enter by direct penetration.* Plant Cell, 2006. **18**(3): p. 731-46.
